# Evaluation of a student participatory, low-intensity program to improve school wellness environment and students’ eating and activity behaviors

**DOI:** 10.1186/s12966-016-0379-5

**Published:** 2016-05-13

**Authors:** Deanna M. Hoelscher, Alicia Moag-Stahlberg, Karen Ellis, Elizabeth A. Vandewater, Raja Malkani

**Affiliations:** University of Texas School of Public Health, Austin Regional Campus, Michael & Susan Dell Center for Healthy Living, 1616 Guadalupe St., Suite 6.300, Austin, TX 78701 USA; Ceres Connections, Ltd., 3818 Louise St., Skokie, IL 60076 USA; MMS Education, 105 Terry Drive, Suite 105, Newtown, PA 18940 USA

**Keywords:** School wellness policies, Obesity, Students, Adolescent, Dietary behaviors, Physical activity, School meals, Health promotion

## Abstract

**Background:**

Most schools have not fully implemented wellness policies, and those that have rarely incorporate meaningful student participation. The aim of the Fuel Up to Play 60 (FUTP60) program is to help schools implement wellness policies by engaging students in activities to improve access to healthful, good tasting food and drinks, and increase the number and type of opportunities for students to be physically active. The aim of this paper is to present initial student-level results from an implementation of FUTP60 in 72 schools, grades 6–9.

**Methods:**

The study used a non-controlled pretest/posttest with serial cross-sectional data. School process data and student-level data were collected in fall 2009 (pre-intervention) and spring 2010 (post-intervention). School wellness practices were captured during a baseline needs assessment survey. Validated self-administered questionnaires assessing dietary and physical activity (PA) behaviors were administered to students in grades 6–9 in the 72 pilot schools. Mixed-effects logistic regression controlling for clustering of schools and demographics was used to calculate odds ratios and confidence intervals to evaluate changes pre- and post- intervention.

**Results:**

All 72 schools implemented FUTP60 during the 2009–2010 school year. Action strategies most frequently chosen by the schools included increasing breakfast participation and new activities before and after school. Positive and significant changes in students’ behaviors (*n* = 32,482 at pretest and 29,839 at post-test) were noted for dairy, whole grains, fruit, and vegetable consumption and PA levels pre- and post-intervention (OR 1.05 to 1.27). Students aware of the program at post-test were significantly more likely to report healthier eating and PA behaviors than students unaware of the program (OR 1.1 to 1.34).

**Conclusions:**

FUTP60 pilot findings indicate that a low intensity program focused on wellness policy implementation is associated with small positive changes in student behaviors, especially when students were aware of the program. Although these initial results are promising, a more rigorous controlled study is warranted as a next step.

## Background

A recent report from the United States (U.S.) Institute of Medicine (IOM) concluded that schools are a crucial focal point for prevention strategies [1]. In particular, schools provide both food and opportunities for physical activity (PA), so the school environment can have a significant effect on student diets and activity levels [2]. One assessment of changes made between the 2006–07 and 2009–10 school years in elementary schools’ food environments was made using a 16-item questionnaire. The authors of this article surmised that the number of schools that had made improvements during this time period was low. The guidelines that were the least likely to be implemented included changing lunch items to include more whole grains and low-fat/non-fat dairy foods and focusing on limiting competitive food sales to fruits, vegetables, whole grains, low-fat dairy, water and 100 % juice [3].

The Healthy, Hunger-Free Kids Act of 2010 strengthened and reinforced the need for school districts’ implementation of wellness policies for nutrition and physical activity in the United States [4]. Unfortunately, a majority of students do not attend schools that have fully implemented wellness policies for nutrition and physical activity [5]. Analysis of surveys from a nationally representative sample of schools identified gaps between schools’ wellness practices and policy requirements. This study found that the percentage of schools that required all five areas of the wellness policy mandate decreased between 2009–10 and 2010–11 school years, from 55 to 46 %, respectively. The authors of the study indicated that the decrease in the number of school districts with competitive food and beverage guidelines was the primary contributor to the overall negative trend [5].

Effective implementation of wellness policies relies on the students’ acceptance of the changes. Yet, most school wellness efforts have overlooked the most crucial stakeholders — the students themselves [6]. The involvement of students has been shown to increase student acceptance in an array of health-related areas and is therefore promising in the area of obesity prevention [7, 8]. Students have valuable, relevant ideas that improve the content of programs and make buy-in from others, including parents, much easier [9, 10]. Studies in school reform found that when students had meaningful involvement in an effort there was improved school performance and a personal connection was fostered between the student, school and staff [11, 12].

The objective of this study was to evaluate implementation of the Fuel Up to Play 60 (FUTP60) program in schools that were diverse in racial/ethnic composition, size, regional location, and socioeconomic status. The program aim is to help schools implement their wellness policies by engaging students in activities to improve access to healthful, good tasting food and drinks, and increase the number and type of opportunities for students to be physically active. The purpose of this paper is to describe the initial implementation of the FUTP60 program and to assess the effects of the program on student nutrition- and physical activity-related behaviors in 72 schools during the 2009–2010 school year.

## Methods

### Study design

This study used an uncontrolled, pre-test/post-test evaluation of a low-intensity intervention, FUTP60, with serial cross-sectional samples. Primary outcome measures to evaluate the effect of school’s implementation of the program included student consumption of fruits, vegetables, whole grains, milk/low fat dairy foods, and physical activity in and outside of school.

### School recruitment

The geographic areas chosen to recruit schools were based on Dairy Council staff availability and a U.S. National Football League (NFL) Club location. To represent different regions of the United States, recruitment focused on districts in the Northeast, Midwest, Northwest, and Southwest (Table 1). The goal was to recruit a convenience sample with a wide variety of school types and students. The school districts recruited represented large and small enrollment, school lunch and breakfast programs availability, a mix of student demographics (race, ethnicity, and socio-economic status), and urban, rural, and suburban settings. One school district in an area without an NFL team was also recruited.

**Table 1:** Fuel Up to Play 60 (FUTP60) school district location, number of schools & students, and school grade levels at baseline data collection (2009)

Cities and states where school districts were located	Schools (*n*)	School grade levels^a^	Total students (*n*)
Philadelphia, Pennsylvania	10	5th–8th	2282
Des Moines, Iowa	8	6th–8th	5234
Chicago, Illinois	4	5th–8th	2950
Minneapolis, Minnesota	3	5th–8th	1182
Newark, New Jersey	9	6th–8th	1750
Houston, Texas	12	6th–8th	9607
Bloomington, Indiana	4	5th–8th	4203
Phoenix, Arizona	7	7th–9th	7904
Meridian, Idaho	12	6th–8th	10173

^a^School grade levels vary in middle school, and can range from

5th grade to 9th grade, depending on the location

School districts and schools were compensated for staff or other resources necessary for data collection. Each participating school had opportunities to apply for grants totaling up to $5000. Schools were given monetary incentives to complete student surveys, among other evaluations, within the necessary timeframe.

### Consent and approval

The district administration for each school district approved implementation of the program and the subsequent evaluation. Parents provided consent for student participation in all school wellness activities using a passive or ‘opt out’ consent process; student assent was required. The Institutional Review Board at The University of Texas Health Science Center at Houston approved study protocols (UT Health, HSC-SPH-15-0590).

### Program description

**Fig. 1:**
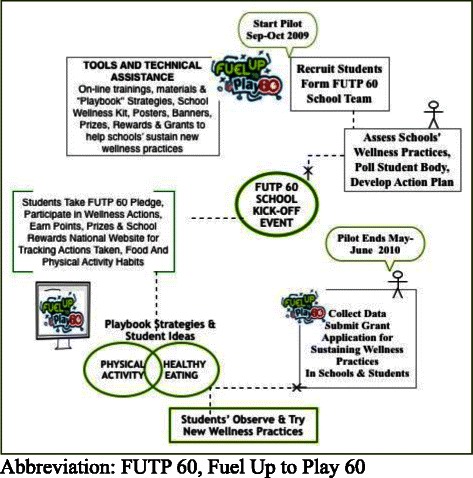
Diagram of Fuel Up to Play 60 Program Elements and the sequence schools followed. School adapted program elements to meet priority needs for school wellness and students’ interests. After schools implemented the program Kick-Off, other activities were done in tandem.

FUTP60 is a low-intensity program without detailed curricula and instructions for delivery. The National Dairy Council (NDC) and National Football League (NFL) created FUTP60 with expert advisors, students, and school stakeholders to include a community participatory model [13, 14] that includes broad engagement by school members, e.g., teachers, parents, school staff, students. The program goals were to increase access and consumption of fruits, vegetables, whole grains and low-fat and fat-free dairy, and to increase opportunities to be active for least 60 minutes every day. Based on school resources, program advisors’ time, and school testing, among other factors, schools determine the intensity or depth of the program’s implementation (Fig. 1). FUTP60 has two main program components: [1] social marketing elements that leverage NFL role models to motivate and engage youth, and [2] a web-based support system for school program leaders.

A Dairy Council staff member served as the implementation study coordinator, who was responsible for several schools in a region. The coordinator’s roles included school district recruitment, coordination of events with NFL teams or players, provision of on-site training, monitoring of program progress, assistance with data collection, and serving as a liaison with national evaluation team members.

The program advisor was the main coordinator of the intervention at the school level, and was responsible for communication with external partners and the implementation study coordinator. The program advisor position was voluntary, and was staffed with teachers, school nurses, physical education and health teachers, and school food service professionals who had the enthusiasm and capacity to serve in this role. In some schools, the principal or other school administrator nominated or appointed the program advisor. The first task of the advisor was to form a school team with interested school-level stakeholders (e.g., school administrators, teachers, school food service professionals, parents, and community representatives) and between 10 to 20 students. In addition to intervention duties, the program advisor at each school completed process evaluation questionnaires that included questions about strategies implemented, program materials and involvement of the students.

The school team was responsible for conducting an initial evaluation of the school wellness environment using the FUTP60 School Wellness Investigation (SWI) survey along with student surveys, which assessed possible changes to food, nutrition, and physical activity and ideas about using NFL merchandise, among other items, as rewards and prizes for students' participation. The SWI instrument was completed by the school team, with input from various school stakeholders (e.g., school nutrition professionals, PE teachers, principals, etc.). The SWI instrument was developed by adapting questions from: (1) the School Health Index [15], which is commonly used for school self-assessment of wellness policies, as well as from (2) a similar survey from the Massachusetts Action for Healthy Kids and the John C. Stalker Institute for Food and Nutrition at Framingham University (“Students Taking Charge”, http://www.actionforhealthykids.org/storage/documents/Students_Taking_Charge/10_minute_student_survey_PDF.pdf). Most changes included adapting questions to ensure that middle school students could understand the content. The instrument included questions about school wellness practices for nutrition (*n* = 13 questions), physical activity (*n* = 10 questions) and family/community involvement (*n* = 6 questions). Schools’ responses were entered into a database and scored based on a four-point rubric; a higher score indicated more wellness practices, or improvements in wellness practices, were in place.

Using these data as an initial needs assessment, schools developed healthy eating and physical activity action strategies to fit their school’s schedule and culture from a menu of resources. FUTP60 provided these resources to aid in implementation in a “Playbook” of best-practice action strategies, ‘how-to’ information for building teams and engaging key audiences, evaluation ideas, grant applications, and videos with NFL players explaining the program and providing inspiring messages to students. All of these were available on the FUTP60 website as well as in hard copy form.

Figure 1 illustrates the flow of the program’s implementation. Once the team had student input and action plans developed, a kick-off event was held for the student body. Schools could also determine if they would participate in the National Challenge. The Challenge required students to earn points by tracking their own actions to improve diet and physical activities during the previous day. Schools also determined the level of promotion used to encourage students to take the wellness pledge located on the FUTP60 website. Technical assistance for adult stakeholders throughout the intervention was delivered in person and via web-based training videos, e-newsletter with tips from educators, and phone and email “help desk.”

An array of NFL merchandise to use incentives or rewards was provided to each school. These items included footballs, t-shirts and jackets, signed small helmets, among others. Schools used grant dollars to purchase other prizes such as mini iPods, gift cards, and movie tickets to incentivize participation. Communication and promotional tools with healthy eating and activity messages included banners, posters, school signage, flyers, template newsletter articles and P.A. announcements within the schools; ideas for kick-off events; NFL player visits; and local challenges and contests with prize opportunities. Local and national public relations increased overall awareness of the program and delivered health-related messages.

### Measures

#### Student behaviors

Students in grades 6–9 at the 72 schools completed a questionnaire adapted from the validated, self-administered School Physical Activity and Nutrition (SPAN) questionnaire [16] at pre-implementation/baseline (September-October, 2009) and post-implementation/follow-up (April-May, 2010). The student self-administered survey included questions about demographics, foods eaten the prior day, physical activity in and outside of school, attitudes about school wellness and FUTP60 program, and participation/awareness of the program.

SPAN questions have been used to assess dietary intake and physical activity behaviors in previous studies, and items have been evaluated in racially/ethnically diverse, low-income populations [17–19], and have been found to have acceptable validity and reliability [18–19]. Dietary assessment items asked about consumption of the food on the previous day: “Yesterday, how many times did you eat….” Categorical response options ranged from none to 3 times. Questionnaire items about breakfast and lunch habits asked if the student ate the school breakfast or school lunch almost always/always, sometimes, or almost never/never. Questions about physical activity asked about on how many of the previous days (range of 0 to 7) that students had engaged in that specific activity. Participation in sports teams at school was assessed with an item that had response options ranging from 0 to 3 or more teams. Program awareness was determined with a question that stated, “Have you seen, read or heard about a program in your school called Fuel Up to Play 60 designed to make your school a healthier place by encouraging you to eat great-tasting/good-for-you foods and get more physical activity?," with a yes/no answer response option.

No identifiers were used on questionnaires, and students not in school on the day the survey was administered were not included in data collection for that time period. Each school’s program advisor was instructed about and led the implementation of the student survey. Most schools chose a portion of class time for students to complete the questionnaire.

#### Process evaluation

Process evaluation measures were conducted throughout the school year. Schools’ plans and progress were tracked and documented. Information collected included what activities schools chose to help change nutrition and physical activity and how schools’ students were involved as strategies were implemented. Evaluation methods included coordinators’ observations of events and action strategies at schools; site visits by a member of the national evaluation team, grant applications and results reported by schools’ program advisors, and quantitative surveys completed by school food service professionals (district and school), program advisors, and Dairy Council coordinators.

#### Data analysis

Dietary and physical activity data were dichotomized into variables that approximated U.S. Dietary Guidelines recommendations and the Physical Activity Guidelines for Americans, respectively. Thus, fruit consumption was dichotomized into variables of consumption of three or more times on the previous day versus 2 or fewer times on the previous day. For physical activity, variables were dichotomized into 60 minutes of physical activity on 5–7 days of the past week or physical activity for 7 days of the past week. Breakfast and lunch consumption were dichotomized into always or almost always eat lunch/breakfast versus sometimes or never/almost never.

Mixed-effects logistic regressions, controlling for school attended, gender, race and grade level, were used to calculate odds ratios and confidence intervals to assess changes between pre- and post-program, while accounting for clustering at the school level. At follow-up, mixed effects logistic regression models with the same covariates were used to assess differences in nutrition and physical activity behaviors between students who reported being aware of the FUTP60 program versus those who reported not being aware of the program. Students were modeled as belonging to one of three ethnic groups: Hispanic, African American, or white/other. The other races, including Native Americans, Asians, and Pacific Islanders, each represented less than 5 % of the sample, and were combined into the white/other category as a result. This method has been used previously with SPAN survey analyses [20].

## Results

### Study sample

Seventy-two middle and K-8 schools encompassing grades 6–9 from 11 school districts participated in the FUTP60 implementation (Table 1). The study population recruited represented a wide variety of schools: small, medium and large enrollment sizes; rural, suburban and urban locations; and diverse race/ethnicity and socio-economic status. School enrollment ranged from 145 to 1,750. Most of the schools (*n* = 59, 82 %) were middle schools, while 15 % (*n* = 11) were junior high schools and 3 % (*n* = 2) were high schools.

### Demographics of sample

The school populations included a significant proportion of Hispanic/Latino and/or African-American students (Table 2). In more than half of the schools, 60 % or more of the students qualified for free or reduced-price school meals (range for schools 25 to 87 %). The student population was evenly divided between boys and girls, and the grade distribution was fairly evenly split among grades 6 to 9 with the least number of students coming from the 9th grade (Table 2). There were no statistically significant differences in the gender or racial/ethnic distributions of the students between baseline and follow-up. As expected, the average age at post-intervention was approximately 6 months higher than pre-intervention.

**Table 2:** Description of Fuel Up To Play 60 (FUTP60) student population in fall 2009 and spring 2010 (*n* = 72 schools)

Student demographics	Fall 2009 (*n* = 32,482)	Spring 2010 (*n* = 29,839)
Race and Ethnicity (%)		
African-American	16.8	17.2
Hispanic	28.6	28.1
White / Other	54.7	54.7
Gender (%)		
Female	50.4	50.0
Male	49.6	50.0
School Grade/Age		
Mean age	12.33 (SD = 1.05)	12.90 (SD = 1.14)*
Grade Distribution (%)		
Grade 6	29.5	31.4
Grade 7	34.1	34.6
Grade 8	31.5	30.4
Grade 9	5.0	3.7

*p < 0.05

### Implementation of program components

Seventy-two schools fully implemented FUTP60 between October 2009 and the end of April 2010. The most frequently chosen Playbook action strategies for improving healthy eating included: increasing school breakfast participation (36 % of schools, *n* = 26) and taste-testing/selecting new foods (28 %, *n* = 20). To improve physical activity, the majority of schools focused on new activities before and after school (44 %, *n* = 32) and daily walking clubs before, during, and after school (44 %, *n* = 32). At pre-test, 61 % (18,823 of 30,992) of students were aware of FUTP60 program activities, compared to 82 % (23,604 of 28,888) at follow-up (*OR* = 2.745, *P* < 0.001). In most schools (97 %) at follow-up, at least 50 % of students were aware of FUTP60 program activities, while in 68 % of schools at least 80 % of students were aware of the program.

### Changes to students’ eating and activity behaviors

Students’ self-reported changes in eating and activity behaviors from baseline to follow-up are presented as odds ratios with 95 % confidence intervals (Table 3). The odds-ratios in Table 3 can be interpreted as the extent of change in student behaviors post-intervention, relative to baseline. Thus, for example, taken as a whole, students were 1.27 times more likely to eat whole-grains 3 or more times per day, and 1.25 times more likely to play on one or more sports team after the intervention than before the intervention. Compared to baseline, students were more likely to report consuming dairy, whole grains, fruit or fruit juice and vegetables at follow-up. More significant changes were found for boys compared to girls, especially for milk and dairy products. The likelihood of dietary changes at follow-up compared to baseline was similar among white and Hispanic/Latino children in the schools, but African American students showed fewer positive changes.

**Table 3:** Dietary and physical activity behaviors in Fuel Up to Play 60 (FUTP60) schools at follow-up (spring 2010, *n* = 29,839) compared to baseline (fall 2009, *n* = 32,482)

	All	Gender		Race/Ethnicity		
		Male	Female	White/Other	Hispanic/Latino	African American
Milk ≥ 2 times/d^a^	1.0^b^ (.96–1.03)^c^	1.05^b^ (1.00–1.10)*	.95^b^ (.90–.99)	.98^b^ (.94–1.02)	1.01^b^ (.95–1.07)	1.03^b^ (.95–1.12)
Dairy ≥ 3 times/d^a^	1.05 (1.02–1.09)*	1.08 (1.03–1.14)*	1.02 (0.97–1.07)	1.04 (1.00–1.09)*	1.08 (1.02–1.15)*	1.02 (0.94–1.10)
Whole Grains ≥ 3 times/d^a^	1.27 (1.21–1.34)*	1.33 (1.25–1.43)*	1.19 (1.10–1.29)*	1.32 (1.23–1.41)*	1.20 (1.09–1.31)*	1.29 (1.15–1.45)*
Fruit or Fruit Juice ≥ 3 times/d^a^	1.10 (1.06–1.14)*	1.10 (1.05–1.15)*	1.10 (1.05–1.15)*	1.13 (1.08–1.18)*	1.05 (0.99–1.12)	1.08 (1.00–1.17)*
Fruit ≥ 3 times/d^a^	1.13 (1.08–1.19)*	1.10 (1.03–1.18)*	1.17 (1.09–1.25)*	1.15 (1.08–1.23)*	1.15 (1.05–1.26)*	1.05 (0.94–1.18)
Vegetables ≥ 2 times/d^a^	1.10 (1.06–1.14)*	1.10 (1.05–1.16)*	1.09 (1.04–1.15)*	1.11(1.07–1.17)*	1.08 (1.01–1.16)*	1.06 (0.97–1.16)
Always or Almost Always Eat Breakfast	0.96 (0.93–0.99)	1.02 (0.98–1.07)	0.90 (0.86–0.95)*	0.93 (0.89–0.97)*	1.01 (0.95–1.07)	1.01 (0.93–1.10)
Always or Almost Always Eat School Lunch	0.85 (0.82–0.88)*	0.83 (0.79–0.87)*	0.86 (0.81–0.90)*	0.87 (0.83–0.92)*	0.78 (0.74–0.84)*	0.86 (0.79–0.94)*
PA^d^ ≥ 60 Minutes At Least 5 of 7 Last Days	1.13 (1.09–1.17)*	1.17 (1.11–1.22)*	1.09 (1.03–1.14)*	1.12 (1.07–1.17)*	1.18 (1.10–1.26)*	1.09 (1.00–1.19)
PA^d^ ≥ 60 Minutes Each of 7 Last Days	1.15 (1.10–1.20)*	1.16 (1.10–1.23)*	1.13 (1.05–1.22)*	1.17 (1.10–1.24)*	1.16 (1.06–1.28)*	1.05 (0.93–1.17)
Participated on ≥ 1 Sports Team at School	1.25 (1.21–1.29)*	1.28 (1.23–1.35)*	1.21 (1.15–1.27)*	1.26 (1.20–1.32)*	1.21 (1.13–1.28)*	1.28 (1.18–1.39)*

^a^Self-reported times student ate foods on previous day.^b^Referents (1.0) are data from baseline (fall 2009). ^c^CI, confidence intervals. Confidence intervals that do not overlap with 1.0 are statistically significant. Asterisks (*) indicate statistically significant likelihoods. ^d^PA, physical activity

The likelihood of engaging in physical activity was significantly higher among all students at follow-up relative to baseline except among African American students, who were no more or less likely to engage in physical activity 60 minutes for each of the last 5 to 7 days. Girls and white/other students were less likely to report always or almost always eating breakfast in spring compared to fall; all groups were less likely to report always or almost always eating school lunch at follow-up.

The likelihood of consuming milk, dairy foods, fruit or fruit juices, vegetables, breakfast and school lunch was significantly increased in students who reported being aware of FUTP60, compared to those who were not aware of the program post implementation. Also, students who were aware of the program were significantly more likely to report engaging in physical activity (Table 4).

**Table 4:** Dietary and physical activity behaviors for students “aware” of Fuel Up to Play 60 (FUTP60) compared to students “not aware” at follow-up (2010)^a^

Dietary and Activity behaviors	Odds ratios (CI)^b^
Milk ≥ 2 times/d^c^	1.23 (1.18–1.28)*^d^
Dairy ≥ 3 times/d^c^	1.18 (1.14–1.23)*
Whole Grains ≥ 3 times/d^c^	1.00 (.95–1.07)
Fruit or Fruit Juice ≥ 3 times/d^c^	1.14 (1.09–1.18)*
Fruit ≥ 3 times/d^c^	1.15 (1.09–1.22)*
Vegetables ≥ 2 times/d^c^	1.23 (1.18–1.28)*
Always or Almost Always Eat Breakfast	1.34 (1.29–1.39)*
Always or Almost Always Eat School Lunch	1.10 (1.05–1.14)*
PA^e^ ≥ 60 Minutes At Least 5 of 7 Last Days	1.33 (1.28–1.39)*
PA^e^ ≥ 60 Minutes Each of 7 Last Days	1.21 (1.15–1.28)*
Participated on ≥ 1 Sports Team at School	1.14 (1.10–1.19)*

^a^Self-reported times student ate foods on previous day. ^b^CI, confidence intervals. Confidence intervals that do not overlap with 1.0 are statistically significant. Asterisks (*) indicate statistically significant likelihoods. ^c^Self-reported times student ate foods on previous day.^d^Referents (1.0) are students who reported not being aware of Fuel Up to Play 60 in spring 2010. ^e^PA, physical activity

## Discussion

The FUTP60 study demonstrated that a low-intensity, flexible approach to environmental changes in schools can lead to small behavioral changes when the student body is aware and engaged in program implementation. Students reported positive changes for several food and physical activity behaviors post-intervention compared to pre-intervention, although the magnitude of the changes was greater in students who reported familiarity with FUTP60. Although the FUTP60 student-adult team was essential to program implementation, the engagement of the majority of students in the majority of schools could be considered a key factor in eliciting behavior changes over a brief time period.

The social marketing elements highlighting the NFL motivated girls’ and boys’ participation. All communications, messages, banners, and public relations highlighted the NFL and tied in with NFL Play 60 advertising during the American football season. Many of the schools had players from the local NFL Club at a school event. Players visits to school included pep rallies, celebrations at the end of the program, and as a reward for meeting a school goal. Players and students often engaged in physical activity during the event.

There is an excitement generated from professional sports that can be influential in promoting health behaviors. Studies have shown that professional sports players have influenced youth with regard to product choices and other buying behaviors [21]. Fuel Up to Play 60 leveraged NFL marketing and communications at a national level to complement school-level communication to motivate students’ involvement and eating and activity behaviors at school. As others have shown, professional sports players are powerful messengers and, partnered with public health, can motivate youth to make healthful choices [22].

When examined by racial/ethnic group, dietary intake and physical activity changes were less likely to reach significance in African American students. Some of the foods that did not show significant changes from pre-test to post-test included milk and dairy products, which might be as a result of perceived lactose intolerance [23]. In terms of physical activity, our data show that African American middle school students were at lower levels of physical activity at baseline, so improvement might be more difficult, especially with a less intensive intervention. These levels of physical activity are in contrast to our other work in Texas, which shows African American middle school students with higher levels of physical activity [24]. Further development work with FUTP60 should focus on addressing barriers to dietary intake or physical activity, either perceived or through additional changes in the school environment, especially focused on strategies that are most effective in African American children.

Breakfast participation was a focal point for improving nutrition practices at 36 % of pilot schools, although increased school breakfast consumption was not one of the stated behavioral goals of the FUTP60 intervention. Students who were aware of FUTP60 were more likely to report always or almost always consuming breakfast post-implementation compared to students who were not aware of the program. This finding is in contrast to the overall post-intervention finding that girls were less likely to always or almost always eat breakfast. In general, data from U.S. school breakfast programs show consumption of school breakfast decreases throughout the school year [25], so it may be that the increased messaging from FUTP60 might need further testing among middle school girls, or that the intervention was not robust enough to overcome the decreases in school meal consumption that are documented from the beginning to the end of the school year in both school breakfast and school lunch [25, 26]. Greater involvement of the students in school food service activities and partnership of students with cafeteria staff can potentially lead to greater participation in both breakfast and lunch consumption over time. In addition, incorporating more activities involving cafeteria staff in the FUTP60 Playbook may be merited.

There are only a few published studies on student participation as a strategy to improve the school wellness environment. Those reviewed in comparison with FUTP60 have differences in program attributes, study design, measurement instruments and outcomes [6, 7, 27]. School-based interventions conducted prior to 2006 were mostly randomized controlled trials implemented over several school years [28–30]. Outcomes of these studies included changes in body fat, weight and/or waist circumference, or school environment changes such as lower fat and sodium content of school meals and optimized physical education classes that lead to an increase in time that students are vigorously active. The majority of these highly controlled interventions had modest changes in outcomes [28–30]. A program like FUTP60 may be a good adjunct to these more intensive programs, reinforcing key messages through the school environment, and having students participate in school changes.

Schools implemented FUTP60 to various degrees contingent on contextual factors such as a district administration commitment, time given to program advisors, budget, and other priorities such as testing. Therefore, FUTP60 was built to accommodate various levels of intensity in its implementation. FUTP60 is a lower intensity program than others that reported similar results. For example, a small study using a youth development approach to improve the school nutrition environment and students’ diet was conducted in five schools over two school years [7]. The study had two intervention and three control schools with students in grades 4–6 (*n* = 360). The intervention included 12 nutrition education classes that included a youth leadership component with 9 students receiving additional leadership training and details on making changes to the nutrition environment. Outcomes included an increase of ½ serving of fruit in intervention students compared to those in control schools [7].

Another study used a community-participatory approach, along with student leadership, as a method for changing students’ behaviors relative to healthy food consumption and amount and effort of physical activity. The study was summarized as being generalizable and easy to disseminate because of the less rigorous approach and focus on capacity building and training [27]. The study found significant changes in physical activity measures, yet no change took place in fruit and vegetable consumption. The concept of low-intensity programs being a better fit for more schools is a priority result based on the study authors’ account [27], which is the fundamental idea behind the approach used by FUTP60.

### Limitations and strengths

Since this study assessed implementation of a community-based program, the design limited the ability to include control schools or to randomize participation in the program; however, stratifying data based on the student self-awareness of FUTP60 provides initial data on nutrition and physical activity behaviors among students who were aware of, or at least minimally exposed to, the intervention strategies and implementation. As with any cross sectional data, these findings do not imply causality, and it could be that students who are more interested in or already engaging in more healthful behaviors are more likely to be aware of a new health-related program.

In terms of program implementation, schools differed in context in many ways, such as administration buy-in, type of school food service, space limitations, time available from the adult program leaders and additional resources added by the school to implement the program (staff, budget, district food and nutrition services) that were not measured. Replication of this study with a more rigorous study design, such as a cluster-controlled trial with schools randomized to either intervention or comparison condition, is warranted as a next step.

The diversity and number of schools added strength to the findings and potential for generalizability. Locations of the schools included suburbs and large urban cities with significant percentage of minority students, different types of school food service operations and differences in administrative support. In addition, student survey measures have been previously used and have demonstrated adequate validity and reliability in diverse student populations.

## Conclusions

Results from the FUTP60 intervention suggest that a low-intensity program that engages students in the implementation of wellness practices can be effective in achieving modest behavior change in various school settings with diverse student populations (grades 6–9). FUTP60 might have a greater impact on student behavior if implemented as an adjunct to, and simultaneous with, more intensive nutrition and physical education programs. Alternately, a program such as FUTP60 might be a good first step for schools or school districts that seek to improve child health, but are faced with multiple barriers that interfere with more intensive programmatic efforts. Further research is needed to understand, with a level of certainty, the degree of effort needed by a school to achieve meaningful changes in the environment and students’ wellness practices.

## Abbreviation

FUTP 60: fuel up to play 60
